# Book Review: Medicinal Plants for Holistic Healing

**DOI:** 10.3389/fphar.2019.01053

**Published:** 2019-09-18

**Authors:** Lyndy J. McGaw, Ajay Kumar Srivastava, Chung-Ho Lin, Vanessa Steenkamp

**Affiliations:** ^1^Phytomedicine Programme, Department of Paraclinical Sciences, Faculty of Veterinary Science, University of Pretoria, Pretoria, South Africa; ^2^Department of Botany, St Xavier’s College, Ranchi, India; ^3^Phytochemistry and Bioremediation, Center for Agroforestry, School of Natural Resources, University of Missouri, Columbia, MO, United States; ^4^Department of Pharmacology, Faculty of Health Sciences, University of Pretoria, Pretoria, South Africa

**Keywords:** medicinal plants, cosmeceutics, skin cancer, skin infections, tuberculosis

It is undeniable that medicinal plants have played, and continue to play, a major role in the lives of people worldwide. This book appropriately opens with a fascinating and concise, though comprehensive, history of traditional medicine and its African heritage, starting approximately 4,000 years ago. The reader is taken on a journey through the use of medicinal plants for specific diseases, which originated from trial and error to the development of modern herbal preparations or drugs with specific treatment targets. The book concludes by focussing on realizing the potential of medicinal plants. In essence, the book comes full circle, portraying global interests and highlighting the importance of medicinal plant usage in health and well-being. The intense pace of today’s lifestyle has led to a dramatic rise in the incidence of many ailments, such as cancer, cardiovascular disease, and diabetes. This book mentions several diseases of present and future significance including cancer, tuberculosis, and skin infections and potential plant-based medications against them. All remedies require varying lead times to enable development, and by then, casualties may rise. However, this book has given a useful background and highlighted research opportunities aimed at maximizing the realization of the potential of plants found in South Africa. Such disease threats include various types of skin cancers, hyper-pigmentation, oral disorders (with changing food and eating styles), and many others. This textbook provides a thorough and detailed description of many classes of plant-derived natural products for medical use, particularly regarding nutraceutical and cosmeceutical applications. Functional foods are foods consumed frequently that have potential health benefits, and nutraceuticals refer to products sold over the counter containing phytochemicals putatively enhancing health. The use of these purportedly beneficial foods and supplements is particularly relevant in the field of cancer prevention, as covered in the second chapter of the book. In this chapter, it is noted that substances found in plants may be able to prevent, inhibit, or even reverse formation of cancerous cells. Skin infections, acne, and aging negatively affect confidence, and plants have been used for centuries as natural cosmeceuticals to enhance beauty and combat infections. The interest of international cosmetics enterprises has been stirred by the possibilities in this sphere. An example mentioned in Chapter 3 is the incorporation of *Mesembryanthemum crystallinum* into an antiaging formulation by a renowned cosmetics company.

Edited by a renowned expert in the field, Professor Namrita Lall of the University of Pretoria, the book comprises a collection of chapters on diverse areas of the potential application of medicinal plants. It covers a variety of topics with a major focus comprising the traditional use of medicinal plants and their potential application. The book consists of 10 insightful and well-written chapters supported by neat and appropriate illustrations. The book has a two pronged approach, with holistic health reflecting on healing from the point of view of traditional healers on the one hand and health and well-being directed at practitioners in orthodox medical fields on the other. Traditional healers have a long history of using medicinal plants and trust their effectiveness, while orthodox medical professionals may view the use of plants in medicine with some scepticism in light of the general paucity of scientific data supporting the safety and efficacy of these remedies. Books such as this, combining traditional knowledge of plants with Western scientific methods evaluating their use in accepted model assay systems, will go a long way toward building bridges between the two modes of thinking.

The body of the book comprises thought-provoking and diverse chapters on the potential for utilization of plants in treating diseases of the skin and use of traditional medicine as anticancer, anti-aging, and anti-infective agents. Chapters on oral care, progressive macular hypomelanosis, and tuberculosis are included, with the useful addition of a chapter on garlic and its many uses. Each topic is introduced by providing a background on the disease, which contains updated statistics on the prevalence thereof, followed by the associated pathology, pharmacologically approved drugs currently on the market used for treatment of the disease, an array of medicinal plants used for treatment accompanied with a list of their active phytoconstituents and chemical structures thereof, as well as scientific evidence for use. Additionally, photographic plates of the most commonly used plants are provided, which serve as useful aids in plant identification. For example, a large number of plant species are used for antiaging properties such as *Aloe vera*, *Aspalathus linearis*, and *Populus nigra*, and photographs of these and many other species are provided. Most likely owing to space contraints, the pictures are small, but the plates illustrate the diversity of plants used to treat the targeted conditions.

The book concludes with an extremely useful chapter on maximizing the value of medicinal plants in terms of commercialization. This is an oft-neglected aspect of medicinal plant research highly deserving of increased attention, as much potential value is locked in plants that have been used for centuries for cosmetic or medicinal treatments. Thus, this book contributes toward bridging the gap between the traditional healer and the modern scientist, as consumers are increasingly combining treatments sourced from both traditional and orthodox spheres. It not only calls for an enhanced understanding and appreciation of medicinal plant usage but also cautions that natural does not imply safe or that no side effects will be experienced, thereby clearly indicating the need for scientific testing to validate efficacy and safety.

Cognisance should be taken of the important role of plants in drug development—they remain sources of treatment that are desperately needed. Emphasis should be placed on integrating the knowledge of traditional healers and verifying activity using modern techniques. The knowledge of traditional healers includes treatment with medicinal plants as well as psychological aspects that cannot be tested in a laboratory. However, scientific techniques involving targeted bioassay screening, synergistic studies, and detailed mechanism of action investigations may go a long way toward assisting with validation of traditional remedies as well as identifying potential future drug leads. Reanalyzing medicinal plants using this perspective could reveal a world of knowledge encompassing valuable potential sources of drugs and hope for future medications for currently incurable diseases.

The work presented in this book will be most advantageous for undergraduate and postgraduate students as well as academic staff researching plants for medicinal and cosmetic purposes in South Africa and indeed the rest of the world. The authors’ unbiased approach in guiding the reader through fundamental chemical principles, modes of action, and product formulation of bioactive natural products for medical applications, ranging from treatment and prevention of skin disorders, infections, and aging to dental disease, makes this a useful reference for students and pharmacognosists, pharmacists, medicinal chemists, phytochemists, dermatologists, and cosmetic chemists. The accessible and informative writing style, which is consistent throughout the chapters, also lends itself to being of interest to lay people with a particular interest in medicinal plants and how they work. The writing is pitched at a level to provide the specialist with intriguing information, updated facts and references but will also resonate with the novice who would like to gain more in-depth information on the highlighted topics.

## Author Contributions

All authors contributed to writing the book review. LM edited the manuscript and submitted it.

## Conflict of Interest Statement

The authors declare that the research was conducted in the absence of any commercial or financial relationships that could be construed as a potential conflict of interest.

